# Effects of nitrogen stress and nitrogen form ratios on the bacterial community and diversity in the root surface and rhizosphere of *Cunninghamia lanceolata* and *Schima superba*

**DOI:** 10.3389/fpls.2023.1240675

**Published:** 2023-10-18

**Authors:** Yanru Wang, Xiaoyu Li, Xiaoqiang Quan, Haiyan Liang, Lidong Wang, Xiaoli Yan

**Affiliations:** College of Forestry, Fujian Agriculture and Forestry University, Fuzhou, China

**Keywords:** nitrogen stress, nitrogen form ratio, root surface and rhizosphere, bacterial community, *Cunninghamia lanceolata*, *Schima superba*

## Abstract

**Background:**

The bacterial communities of the root surface and rhizosphere play a crucial role in the decomposition and transformation of soil nitrogen (N) and are also affected by soil N levels and distribution, especially the composition and diversity, which are sensitive to changes in the environment with high spatial and temporal heterogeneity of ammonium N (NH_4_^+^-N) and nitrate N (NO_3_^-^-N).

**Methods:**

One-year-old seedlings of *Cunninghamia lanceolata* and *Schima superba* were subjected to N stress (0.5 mmol L^-1^) and normal N supply (2 mmol L^-1^), and five different N form ratios (NH_4_^+^-N to NO_3_^-^-N ratio of 10:0, 0:10, 8:2, 2:8, and 5:5) were created. We analyze the changes in composition and diversity of bacteria in the root surface and rhizosphere of two tree species by high-throughput sequencing.

**Results:**

Differences in the composition of the major bacteria in the root surface and rhizosphere of *C.lanceolata* and *S. superba* under N stress and N form ratios were not significant. The dominant bacterial phyla shared by two tree species included Proteobacteria and Bacteroidota. Compared to normal N supply, the patterns of diversity in the root surface and rhizosphere of two tree species under N stress were distinct for each at five N form ratios. Under N stress, the bacterial diversity in the root surface was highest at NH_4_^+^-N to NO_3_^-^-N ratio of 10:0 of *C. lanceolata*, whereas in the root surface, it was highest at the NH_4_^+^-N to NO_3_^-^-N ratio of 0:10 of *S. superba*. The NH_4_^+^-N to NO_3_^-^-N ratio of 5:5 reduced the bacterial diversity in the rhizosphere of two tree species, and the stability of the bacterial community in the rhizosphere was decreased in *C. lanceolata*. In addition, the bacterial diversity in the root surface was higher than in the rhizosphere under the N stress of two tree species.

**Conclusion:**

The bacterial compositions were relatively conserved, but abundance and diversity changed in the root surface and rhizosphere of *C. lanceolata* and *S. superba* under N stress and different N form ratios. The heterogeneity of ammonium and nitrate N addition should be considered for N-stressed environments to improve bacterial diversity in the rhizosphere of two tree species.

## Introduction

Nitrogen (N) is one of the nutrient elements necessary for the normal functioning of the plant organism, and it can affect the metabolism and resource allocation of plants. It has become the main factor restricting plant growth in terrestrial ecosystems (Veresoglou et al., 2012). The main inorganic N that can be directly absorbed and utilized by plants are ammonium nitrogen (NH_4_^+^-N) and nitrate nitrogen (NO_3_^−^-N), which have strong temporal fluctuations and spatial heterogeneity in forest soils. In particular, the soil N conversion process makes their content and distribution highly heterogeneous (Zhou et al., 2013; Carvalho et al., 2015; Xiao et al., 2017). As N deficiency and heterogeneous distribution environments are prevalent in nature, plants have developed a variety of adaptive mechanisms to respond to N stress and regulatory strategies for the uptake and utilization of different N forms during long-term evolution (Nacry et al., 2013; Kiba and Krapp, 2016). One of the most important response strategies is to coordinate the microorganisms in the plant rhizosphere to adapt to the N-deficient environment to promote plant growth (Choudhary et al., 2016). In addition, an important study has shown that the root compartment consists mainly of the inner root layer, the root surface, and the rhizosphere. Different root compartments have different microbiomes (Edgar, 2013; Zgadzaj et al., 2016). Therefore, studying the differences in bacterial communities between the root surface and rhizosphere under different N supply levels and N form ratios is helpful to reveal the responses of core bacteria in the root compartment to N deficiency and heterogeneous distribution environments.

Soil microorganisms play an important role in converting insoluble minerals into forms available to roots due to their rapid reproduction, large numbers, and high metabolic capacity. Moreover, they are also extensively involved with the soil N cycle and have an important influence on biogeochemical cycles (Jing et al., 2015; Dini-Andreote et al., 2016). Bacteria are one of the most essential taxa of soil microorganisms. They are extremely sensitive to changes in the nutrient environment, especially the N content of the soil around roots, which is one of the most important factors influencing soil microbial diversity and community structure (Kavamura et al., 2018; Chen et al., 2019; Li et al., 2020). Excessive N addition significantly suppressed the abundance and diversity of the bacterial community, especially the abundance of N-fixing bacteria (Berthrong et al., 2014; Zeng et al., 2016; Zhang, 2003). Simultaneously, the rhizosphere bacteria help plants uptake N and indirectly contribute to the maintenance of mineral–nutrient balance (Bai et al., 2022). In addition, it has been found that soil ammonium N effectiveness may also lead to changes in bacterial richness. The bacterial communities are significantly correlated with soil nitrate N content under N deficiency in wheat, and N deficiency significantly inhibited the propagation of ammonia-oxidizing microorganisms such as the Nitrospirae phylum (Xiong et al., 2022). Acidic bacteria had different response characteristics to NH_4_^+^-N and NO_3_^−^-N. The abundance of acidic bacteria under the NH_4_^+^-N treatment was higher than that under the NO_3_^−^-N treatment, but their abundance decreased with the increase of NH_4_^+^-N (Zhao et al., 2015; Guo J. et al., 2021). Therefore, exploring the effects of different N form ratios of ammonium and nitrate on bacterial communities is key to understanding soil microbial diversity.

As the main silvicultural species in the subtropical region of China, *Cunninghamia lanceolata* and *Schima superba* occupy an important position in China’s southern forestry. More than 20 million ha of forest plantations are made of the fast-growing conifers of *C. lanceolata* in southern China. In recent years, the problems of pure forestation and multi-generational succession of *C*. *lanceolata* have resulted in decreasing soil fertility, lower stand yield, and low ecological service function, which have seriously affected their sustainable management and development (Tian et al., 2011; Liu et al., 2018; Suo et al., 2019; Fei et al., 2020). NH_4_^+^-N and NO_3_^−^-N deficiency is one of the major factors limiting the productivity of *C. lanceolata* and *S. superba* plantations. It is particularly important to optimize planting under N deficiency and heterogeneous distribution environments to balance the economic and ecological benefits of forest plantations. Studies have shown that *C. lanceolata* preferred the uptake of ammonium N, while *S. superba* preferred the uptake of nitrate N (Yan et al., 2020). Selecting broadleaf species with different ecological strategies to create mixed forests can reduce the negative effects of planting artificial coniferous forests and pure forests of fast-growing species. Research on the microbial communities in the root surface and rhizosphere of different tree species needs to be intensified, although the importance of appropriate mixing is widely recognized.

At present, there are few reports on the effects of N stress and form ratios on bacterial communities in the root surface and rhizosphere of *C*. *lanceolata* and *S*. *superba*, especially because the effects of different N form ratios on bacterial communities are relatively scarce. Most of the previous studies on bacterial communities have focused on the rhizosphere and ignored the role of the root surface at the critical interface between the plant and the soil. In addition, the relationship between the composition and diversity of bacterial communities in the root surface and rhizosphere in response to different N form ratios under N stress is not clear. In summary, under the background of increased soil N stress and a highly heterogeneous distribution environment, we investigated the similarities and differences in the composition and diversity of bacteria in the root surface and rhizosphere of *C. lanceolata* and *S. superba*. The aim is to provide comprehensive insights into the rational mixing of *C. lanceolata* and *S. superba.* And to provide a scientific basis for rational fertilization of ammonium N and nitrate N for the two major tree species and improve soil N use efficiency under N deficiency environments.

## Materials and methods

### Plant material

In April 2022, the sand culture experiment was conducted in a light-permeable and well-ventilated greenhouse at Fujian Agriculture and Forestry University. One-year-old seedlings of *C*. *lanceolata* and *S*. *superba* were selected, which had uniform growth and were free from pests and diseases. The average height of *C. lanceolata* seedlings was 22.3 cm, and the average ground diameter was 3.73 mm, while the average height of *S. superba* seedlings was 18.5 cm, and the average ground diameter was 2.76 mm. Using washed river sand as the potting substrate, the sand was repeatedly washed with distilled water until the N content in the sand was close to zero (Wu et al., 2011; Yan and Ma, 2021). The washed river sand was sterilized by autoclave at 120°C for 30 min and then packed into plastic pots after cooling. The plastic pots have a diameter of 22.5 cm and a height of 24.7 cm. Each pot was filled with an equal amount of about 50 g of bacterial soil (The tested bacterial soil was mainly Balloon Moses, and each 10 g of bacterial soil contained 120–150 spores). The roots were dipped into the bacterial soil by the root dipping method, and the rest of the bacterial soil was evenly distributed around the roots. The bacterial soil was provided by Gansu Bofeng Agriculture, Forestry, and Animal Husbandry Technology Co, Ltd. (Wuwei, Gansu Province, China).

### Experimental design and culture of the seedlings

In the experiment, two levels of N supply were set up. The total N supply in each treatment was 0.5 and 2.0 mmol L^−1^, respectively, representing N stress (N_1_) and normal N supply (N_2_), where the normal N supply of 2.0 mmol L^−1^ referred to the results of previous studies (Zhang et al., 2006; Zhang et al., 2013). Five different ratios (NH_4_^+^-N to NO_3_^−^-N ratio of 10:0, 0:10, 8:2, 2:8, and 5:5) were created with two N forms (NH_4_^+^-N and NO_3_^−^-N) labeled as *R*_1_, *R*_2_, *R*_3_, *R*_4_, and *R*_5_, respectively (**Table 1**), of which 5:5 is homogenous N supply. Each pot contained one seedling. There were three replicate pots for each treatment, and the total number of pots was 60. NH_4_^+^-N was supplied as (NH_4_)_2_SO_4_, while NO_3_^−^-N was supplied as NaNO_3_, and the concentrations of macroelements (Hoagland formulation) and micronutrients (Amon formulation) were kept the same in the nutrient solution of each treatment, except for the different ratios of NH_4_^+^-N and NO_3_^−^-N concentrations. The pH of the nutrient solution was maintained at 5.5. To prevent the conversion of NH_4_^+^-N from being converted to NO_3_^−^-N, the nitrification inhibitor dicyandiamide (C_2_H_4_N_4_) was added at 7 μ mol L^−1^ to the nutrient solution (Sun et al., 2015), while NaCl was used to adjust the difference of Na^+^ in each treatment by adjusting the nutrient solution with 2.0 mol L^−1^ NaOH and HCl solution (Liang et al., 2022). Each treatment was watered with equal amounts of pure water every 2 days and 50 ml of nutrient solution every 5 days. The experimental process has lasted a total of 180 days.

**Table 1:** Treatments with different N supply levels and form ratios.

N supply level (mmol L^−1^)	NH_4_^+^/NO_3_^−^									
	10:0		0:10		8:2		2:8		5:5	
	NH_4_^+^	NO_3_^−^	NH_4_^+^	NO_3_^−^	NH_4_^+^	NO_3_^−^	NH_4_^+^	NO_3_^−^	NH_4_^+^	NO_3_^−^
N stress	0.5	0	0	0.5	0.4	0.1	0.1	0.4	0.25	0.25
Normal N	2.0	0	0	2.0	1.6	0.4	0.4	1.6	1.0	1.0

### Soil sample collection

At the end of the experimental treatment, the entire seedlings of *C. lanceolata* and *S. superba* were dug up. As a sample of rhizosphere bacteria, the sandy soil attached to the root system was carefully shaken off and placed in sterile bags. As a sample of root surface bacteria, the root tip and the sandy soil still attached to the root system after shaking were also placed in sterile bags. In addition, samples from the same treatment were pooled into one sample and well-labeled. The samples were snap-frozen in liquid N and then stored in an ultra-low-temperature refrigerator at −80°C.

### DNA extraction and MiSeq sequencing

The sample DNA was used as a template to complete the genomic DNA extraction. The Miseq library was then constructed and sequenced after PCR amplification of the bacterial 16S ribosomal coding sequence. The amplification primers were 338F: (5′-ACTCCTACGGGAGGCAGCAG-3′) and 806R: (5′-GGACTACHVGGGTWTCTAAT-3′) for the V3~V4 region. PCR extension reaction system (25 μL): 30 ng DNA sample, 1 μL Forward Primer (5uM), 1 μL Reverse Primer (5uM), 3 μL BSA (2 ng μL^−1^), 12.5 μL 2× Taq Plus Master Mix, and 7.5 μL ddH_2_O. PCR amplification reaction: predenaturation at 94°C for 5 min, denaturation at 94°C for 30 s, annealing at 50°C for 30 s, extension at 72°C for 60 s, 30 cycles, and extension at 72°C for 7 min. The purification effect of PCR products was detected by 1% agarose gel electrophoresis, and the pair-end (PE) double-end sequence data were read using Miseq splicing software. The measured Fastq data were quality controlled and filtered to finally obtain high-quality Fasta data. The DNA extraction and sequencing services were entrusted to Ovation Gene Technology Co. The original sequencing data were deposited in the NCBI SRA database under the accession number PRJNA986907.

### Statistical analysis of data

The obtained multiple sequence clustering operational taxonomic units, OTUs, were analyzed for OTU abundance, Chao1 index, and Shannon index using QIIME 1.8.0. The Chao1 index was calculated as Schao1 = Sobs + *n*_1_(*n*_1_ − 1)/2(*n*_2 +_ 1), where Schao1 is the estimated number of OTUs, Sobs is the observed number of OTUs, *n*_1_ is the number of OTUs with only one sequence, and *n*_2_ is the number of OTUs with only two sequences. The Shannon index was calculated as *H* = −∑(Pi) (ln Pi), where Pi is the proportion of individuals belonging to the species in the sample (Edgar, 2013; Miller et al., 2016; Rognes et al., 2016). Statistical analysis was performed using R software for graphing, and differences between samples were analyzed based on PLS-DA. Differences between N supply levels, root compartments, and tree species were tested using an independent samples *t*-test, and the differences between five N form ratios at the same N supply level were tested using a one-way ANOVA (Duncan’s test, *p* = 0.05), and a four-way ANOVA was used to test the significance of comparisons. The above statistical analyses were performed using SPSS 25.0. Histograms were plotted using Origin 2019 software.

## Results

### Sequencing quality control and significance test

Using a series of sequence quality control procedures: screening, filtering, preclustering process, and chimera removal, 2,599,569 and 2,304,608 sequences were obtained for the root surface and rhizosphere of *C. lanceolata*, with an average of 86,652.3 and 76,820.27 sequences per sample, respectively. In total, 2,979,235 and 2,400,314 sequences were obtained for the root surface and rhizosphere of *S. superba*, with an average of 99,307.83 and 80,010.47 sequences per sample, respectively. The mean coverage values were in the range of 0.94~0.96 for the root surface and rhizosphere of the two tree species (**Table 2**), indicating that the library coverage of the four samples and the confidence level of the bacterial community structure were high.

**Table 2:** Coverage of the bacterial community in the root surface and rhizosphere of *C*. *lanceolata* and *S*. *superba* at different N supply levels and form ratio treatments.

Tree species	Root compartments	N supply level	NH_4_^+^/NO_3_^−^				
			10:0	0:10	8:2	2:8	5:5
*C. lanceolata*	Root surface	N1	0.94 ± 0.01Ab	0.95 ± 0.01Aa	0.96 ± 0.01Aa	0.96 ± 0.00Aa	0.96 ± 0.01Aa
		N2	0.96 ± 0.00Ba	0.95 ± 0.00Ab	0.95 ± 0.01Aab	0.95 ± 0.01Aab	0.95 ± 0.00Ab
	Rhizosphere	N1	0.96 ± 0.01Aa	0.96 ± 0.00Aa	0.97 ± 0.01Aa	0.96 ± 0.00Aa	0.96 ± 0.02Aa
		N2	0.95 ± 0.00Aa	0.96 ± 0.00Aa	0.96 ± 0.00Aa	0.96 ± 0.01Aa	0.95 ± 0.01Aa
*S. superba*	Root surface	N1	0.95 ± 0.01Aa	0.96 ± 0.01Aa	0.96 ± 0.00Aa	0.96 ± 0.00Aa	0.95 ± 0.01Aa
		N2	0.95 ± 0.00Aa	0.96 ± 0.00Aa	0.95 ± 0.01Aa	0.95 ± 0.00Aa	0.96 ± 0.02Aa
	Rhizosphere	N1	0.97 ± 0.01Aa	0.96 ± 0.00Aa	0.96 ± 0.01Aa	0.96 ± 0.00Aa	0.97 ± 0.01Aa
		N2	0.96 ± 0.01Aa	0.96 ± 0.00Aa	0.96 ± 0.01Aa	0.96 ± 0.00Aa	0.96 ± 0.00Aa

Different capital letters indicate the significant difference between the N supply level at the same N form ratio, and different lowercase letters indicate the significant difference between the five N form ratios at the same N supply level (*P* < 0.05).

OTUs were grouped and divided at 97% similarity, and a multifactorial ANOVA was performed on the number of bacterial OTUs and diversity indices (**Table 3**). NH_4_^+^/NO_3_^−^ (*R*), root compartment (*C*), tree species (*T*), *N* × *R*, *N* × *C*, *N* × *T*, *R* × *C*, *R* × *T*, *C* × *T*, *N* × *R* × *C*, *N* × *R* × *C*, and *N* × *R* × *T*, *R* × *C* × *T*, and *N* × *R* × *C* × *T* interactions had highly significant effects on the bacterial OTUs in root surface and rhizosphere, Chao1 index, and Shannon index of the two tree species. N supply level (*N*), and *N* × *C* × *T* interaction had significant effects on the OTU and Chao1 index of the bacterial community, but not on the Shannon index. *N* × *T* interaction had no significant effect on OTU, Chao1 index, and Shannon index.

**Table 3:** Four-way ANOVA of the effects of N supply level, N form ratio, root compartment, and tree species on the OTU, Chao1 index, and Shannon index.

	OTU		Chao1		Shannon	
	*F*	*p*-values	*F*	*p*-values	*F*	*p*-values
N supply level (*N*)	6.704	0.011^*^	40.788	<0.001^***^	0.641	0.426NS
NH_4_^+^/NO_3_^−^ (*R*)	14.436	<0.001^***^	10.836	<0.001^***^	16.723	<0.001^***^
Root compartment (*C*)	791.652	<0.001^***^	787.379	<0.001^***^	661.540	<0.001^***^
Tree species (*T*)	194.556	<0.001^***^	202.596	<0.001^***^	59.999	<0.001^***^
*N* × *R*	17.445	<0.001^***^	19.802	<0.001^***^	17.047	<0.001^***^
*N* × *C*	43.144	<0.001^***^	69.300	<0.001^***^	7.666	0.007^**^
*N* × *T*	1.079	0.302NS	2.125	0.149NS	0.242	0.624NS
*R* × *C*	7.528	<0.001^***^	9.885	<0.001^***^	20.782	<0.001^***^
*R* × *T*	15.704	<0.001^***^	16.195	<0.001^***^	4.274	0.003^**^
*C* × *T*	33.853	<0.001^***^	16.923	<0.001^***^	53.452	<0.001^***^
*N* × *R* × *C*	18.435	<0.001^***^	25.510	<0.001^***^	23.607	<0.001^***^
*N* × *R* × *T*	6.603	<0.001^***^	4.368	0.003^**^	11.075	<0.001^***^
*N* × *C* × *T*	7.780	0.007^**^	32.911	<0.001^***^	1.917	0.170NS
*R* × *C* × *T*	15.618	<0.001^***^	25.733	<0.001^***^	2.776	0.032^*^
*N* × *R* × *C* × *T*	16.288	<0.001^***^	14.140	<0.001^***^	11.441	<0.001^***^

Significance of analysis of variance factor: NS, not significant; *, *p* < 0.05; **, *p* < 0.01; ***, *p* < 0.001.

### Effects of N stress and N form ratios on bacterial OTUs in the root surface and rhizosphere of *C. lanceolata* and *S. superba*

**Figure 1:**
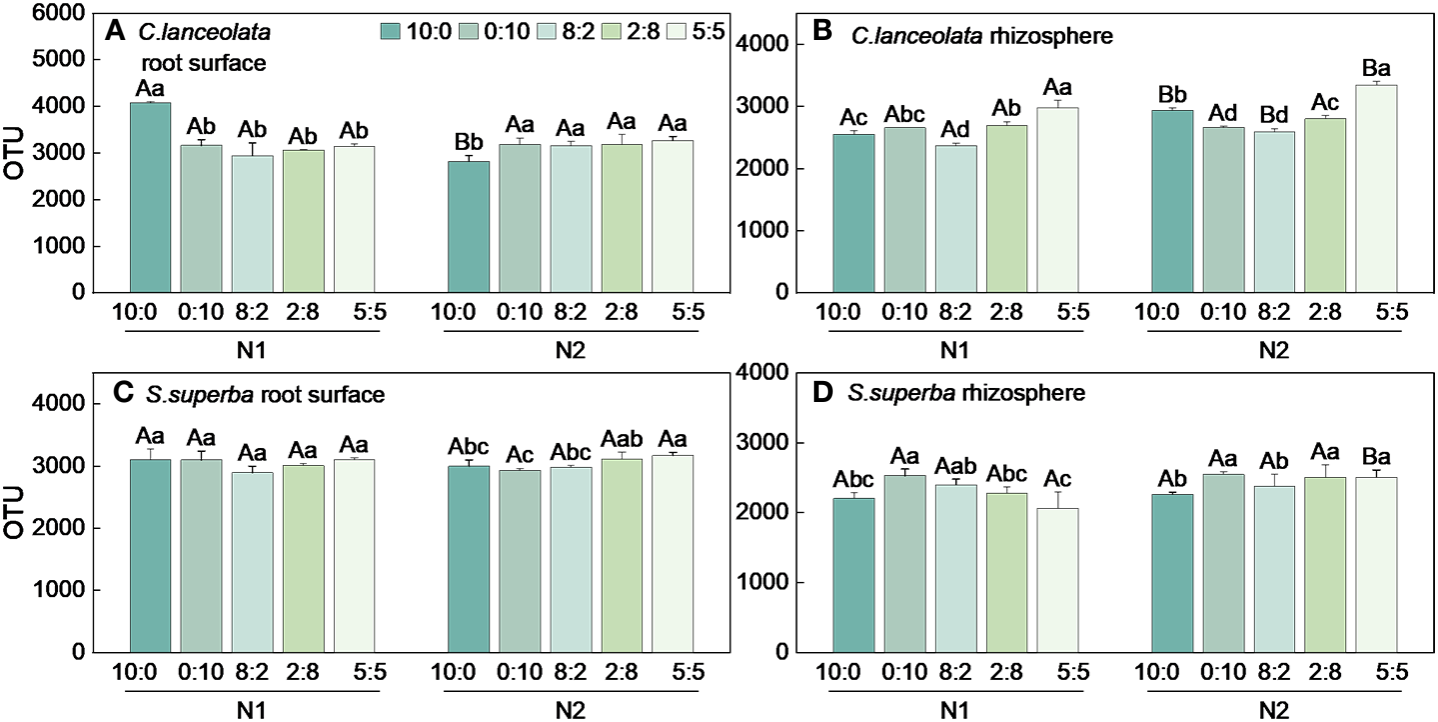
Number of bacterial OTUs in the root surface and rhizosphere of *(C)* lanceolata **(A, B)** and *S. superba* **(C, D)** at different N supply levels and N formratio treatments. Different capital letters indicate the significant difference between the two N supply levels at the same N form ratio, and differentlowercase letters indicate the significant difference between the five N form ratios at the same N supply level (*p* < 0.05).

**Figure 2:**
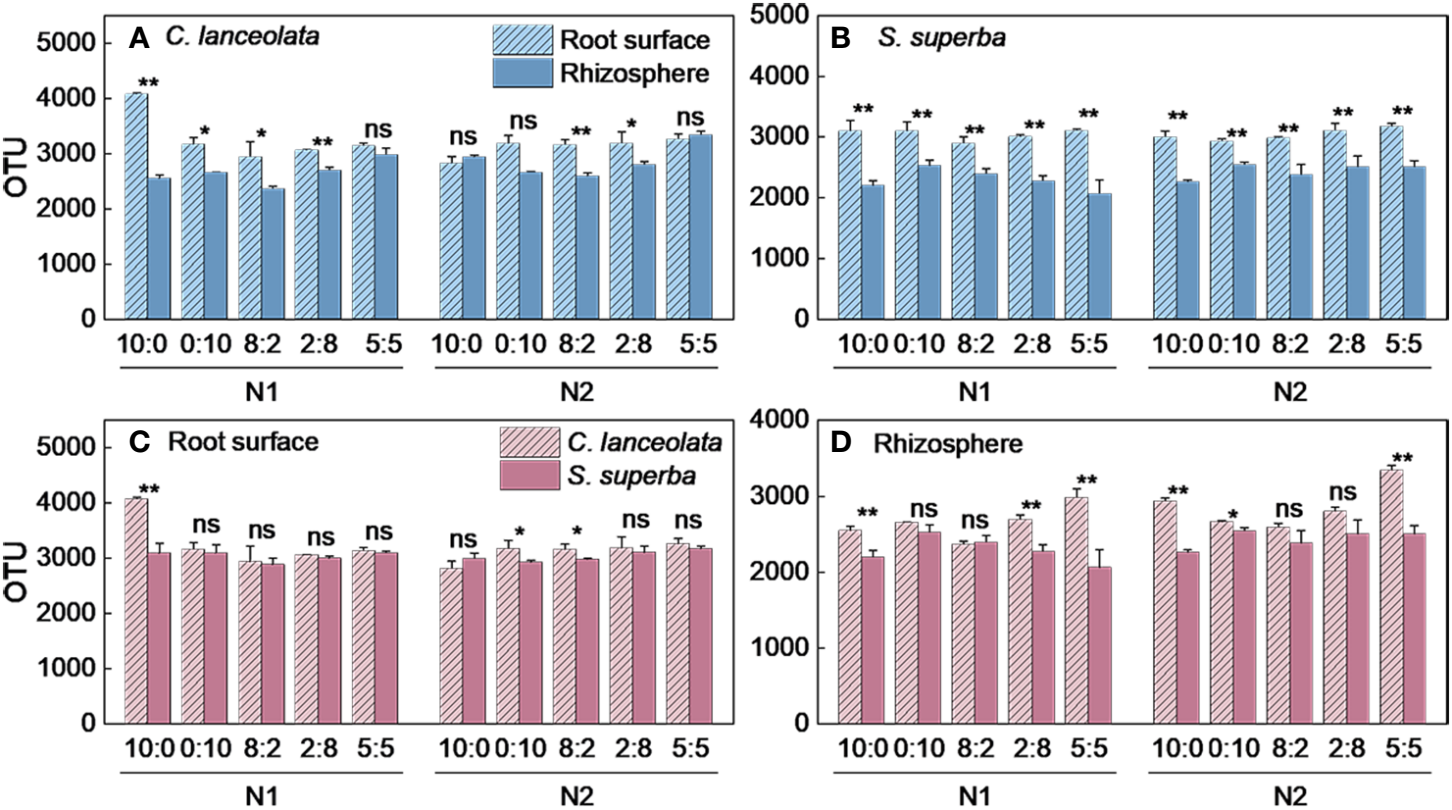
Number of bacterial OTUs in the root surface and rhizosphere of *C. lanceolata* and *S. superba* at different N supply levels and N form ratio treatments. **, *p* = 0.01 and *, *p* = 0.05 indicate significant differences between root compartments **(A, B)** and tree species **(C, D)** at the same N supply level and N form ratio treatments. ns, not significant.

When the NH_4_^+^-N to NO_3_^−^-N ratio is 10:0, the OTU number in the root surface of *C. lanceolata* under N stress was significantly higher than normal N supply and significantly higher than that of the other four N form ratios (**Figure 1A**). Under N stress, the OTU number in the rhizosphere of *C. lanceolata* was lower than normal N supply at all five N form ratios, and the OTU number under two N supply levels showed the lowest at the NH_4_^+^-N to NO_3_^−^-N ratio of 8:2 and the highest at the NH_4_^+^-N to NO_3_^−^-N ratio of 5:5 (**Figure 1B**). The OTU number in the root surface of *C. lanceolata* was higher than in the rhizosphere at all five N form ratios under N stress (**Figure 2A**).

When the NH_4_^+^-N to NO_3_^−^-N ratio of 10:0 and 0:10, the OTU number in the root surface of *S*. *superba* under N stress was significantly higher than normal N supply, and the OTU number was highest at the NH_4_^+^-N to NO_3_^−^-N ratio of 5:5 at two N supply levels (**Figure 1C**). When the NH_4_^+^-N to NO_3_^−^-N ratio is 8:2, the OTU number in the rhizosphere of *S*. *superba* under N stress was significantly higher than the normal N supply. The OTU number was highest at the NH_4_^+^-N to NO_3_^−^-N ratio of 0:10 under two N supply levels (**Figure 1D**). The OTU number in the root surface was higher than in the rhizosphere of *S. superba* under all treatments (**Figure 2B**).

**Figure 3:**
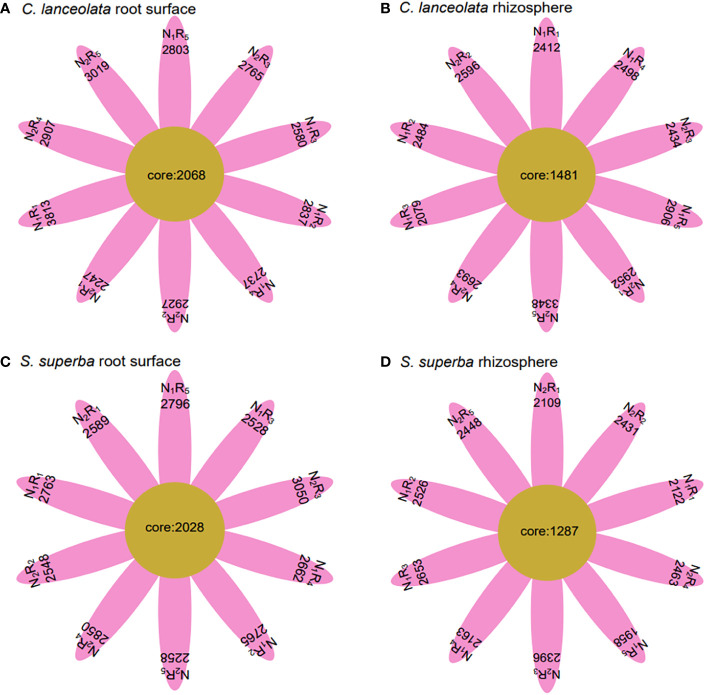
Number of unique and shared bacterial OTUs in the root surface and rhizosphere of (*C*) *lanceolata* **(A, B)** and *S. superba* **(C, D)** at different N supply levels and N form ratio treatments.

Under N stress, the OTU number in the root surface of *C. lanceolata* was higher than that of *S. superba* at all five N form ratios, but the OTU number in the rhizosphere of *C. lanceolata* was higher than that of *S. superba* at most N form ratios (**Figures 2C, D**). The OTU number shared by root surface and rhizosphere of *C. lanceolata* and *S. superba* under all treatments showed the root surface of *C. lanceolata* > root surface of *S. superba* > rhizosphere of *C. lanceolata* > rhizosphere of *S. superba* (**Figure 3**).

### Effects of N stress and N form ratios on bacterial α-diversity in the root surface and rhizosphere of *C. lanceolata* and *S. superba*

**Figure 4:**
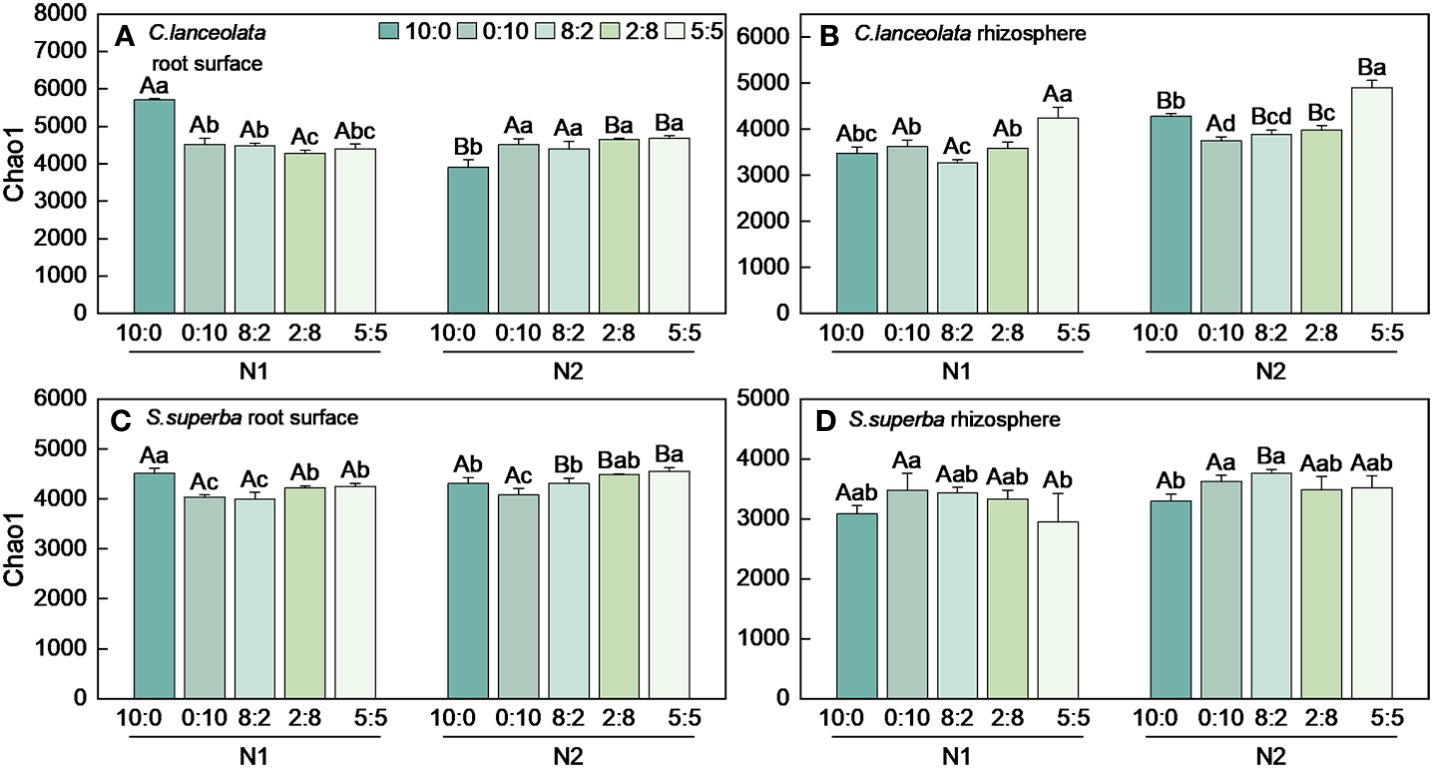
Number of bacterial Chao1 index in the root surface and rhizosphere of *C*. *lanceolata* **(A, B)** and *S. superba* **(C, D)** at different N supply levels and N form ratio treatments. Different capital letters indicate the significant difference between the two N supply levels at the same N form ratio, and different lowercase letters indicate the significant difference between the five N form ratios at the same N supply level (*p* < 0.05).

**Figure 5:**
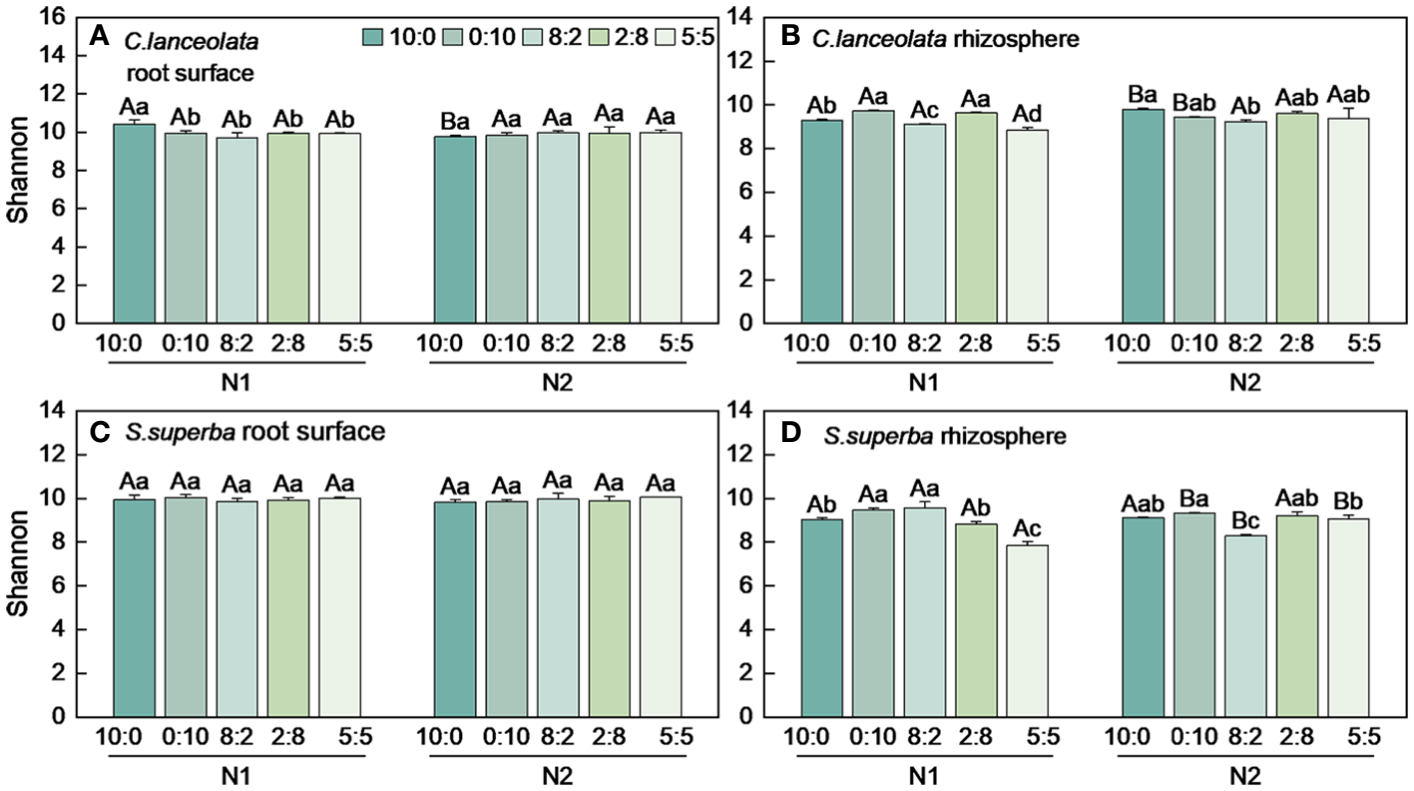
Number of bacterial Shannon index in the root surface and rhizosphere of *C. lanceolata* **(A, B)** and *S. superba (C, D)* at different N supply levels and N form ratio treatments. Different capital letters indicate the significant difference between the two N supply levels at the same N form ratio, and different lowercase letters indicate the significant difference between the five N form ratios at the same N supply level (*p* < 0.05).

**Figure 6:**
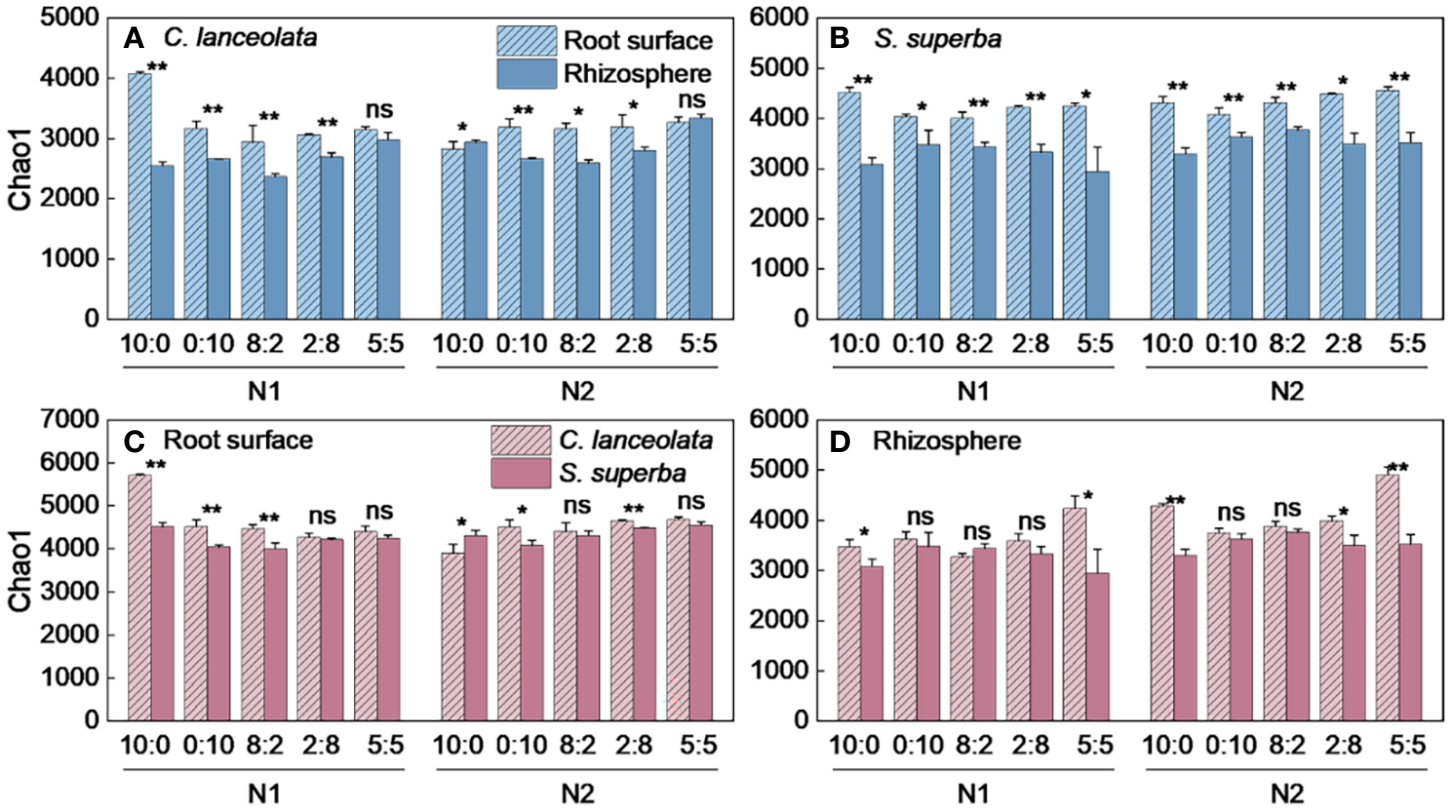
Number of bacterial Chao1 index in the root surface and rhizosphere of *C. lanceolata* and *S. superba* at different N supply levels and N form ratio treatments. **, *p* = 0.01 and *, *p* = 0.05 indicate significant differences between root compartments **(A, B)** and tree species **(C, D)** at the same N supply level and N form ratio treatment; ns, not significant.

**Figure 7:**
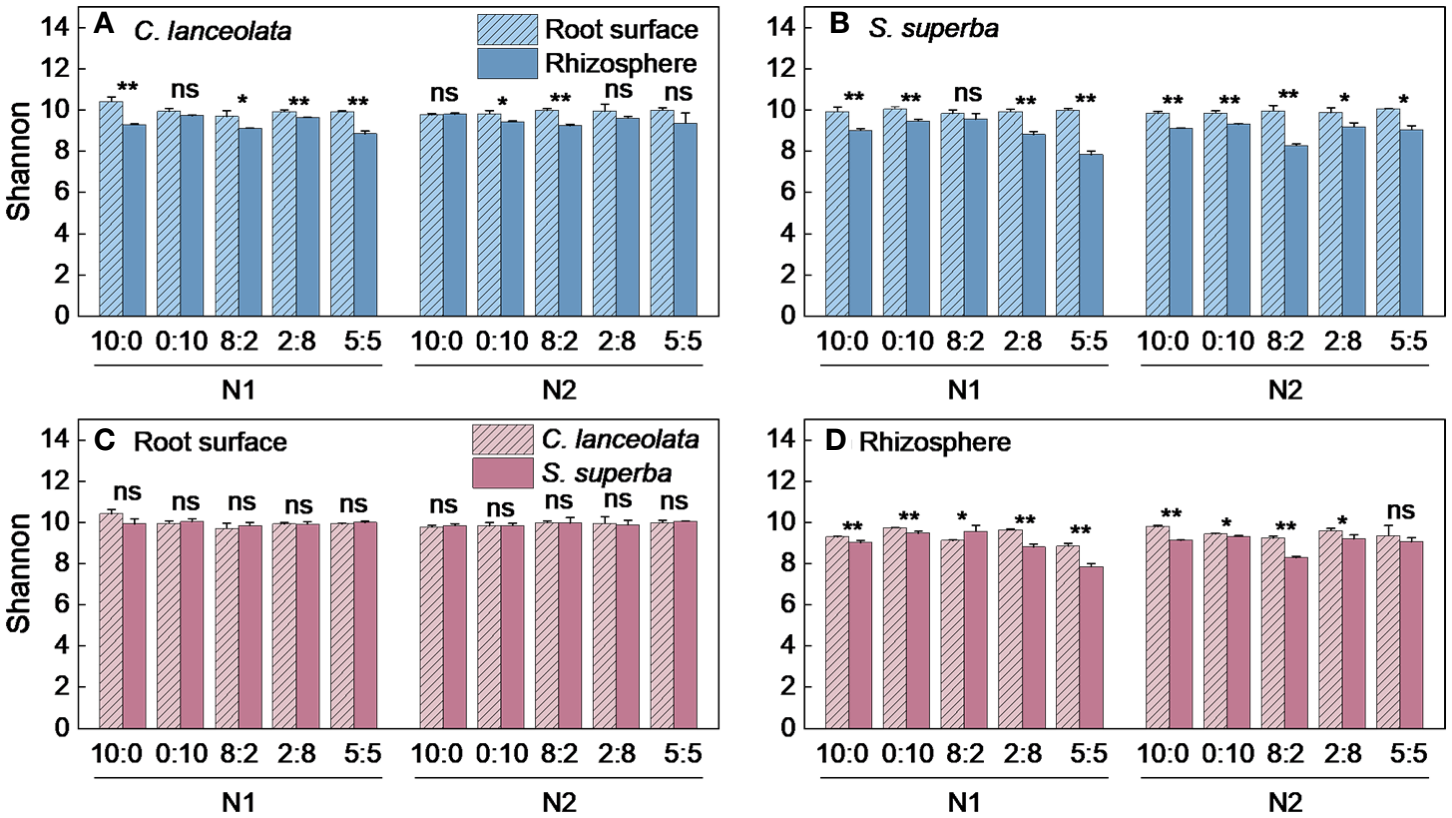
Number of bacterial Shannon index in the root surface and rhizosphere of *C. lanceolata* and *S. superba* at different N supply levels and N form ratio treatments. **, *p* = 0.01 and *, *p* = 0.05 indicate significant differences between root compartments **(A, B)** and tree species **(C, D)** at the same N supply level and N form ratio treatment; ns, not significant.

Both Chao1 and Shannon indices in the root surface of *C. lanceolate* under N stress were highest at the NH_4_^+^-N to NO_3_^−^-N ratio of 10:0, while it was highest at the NH_4_^+^-N to NO_3_^−^-N ratio of 5:5 under normal N supply (**Figures 4A**, **5A**). The Chao1 index in the rhizosphere of *C. lanceolata* under two N supply levels was highest at the NH_4_^+^-N to NO_3_^−^-N ratio of 5:5, while the Shannon index was lowest at this ratio (**Figures 4B**, **5B**). The Chao1 index in the root surface of *C. lanceolata* was higher than in the rhizosphere at all five N form ratios under N stress (**Figure 6A**), and the Shannon index in the root surface of *C. lanceolate* was larger than in the rhizosphere under all treatments (**Figure 7A**).

Under N stress, the Chao1 index in the root surface of *S. superba* was highest at the NH_4_^+^-N to NO_3_^−^-N ratio of 10:0, and the Shannon index was highest at the NH_4_^+^-N to NO_3_^−^-N ratio of 0:10. However, both the Chao1 and the Shannon indices in the root surface of *S. superba* were highest at the NH_4_^+^-N to NO_3_^−^-N ratio of 5:5 under normal N supply (**Figures 4C**, **5C**). The Chao1 index in the rhizosphere of *S. superba* under N stress was lower than normal N supply at all five N form ratios, and the Shannon index showed that N stress was significantly higher than normal N supply at the NH_4_^+^-N to NO_3_^−^-N ratio of 0:10 and 8:2. Both the Chao1 and Shannon indices in rhizosphere of *S. superba* under N stress were lowest at the NH_4_^+^-N to NO_3_^−^-N ratio of 5:5 (**Figures 4D**, **5D**). In addition, both the Chao1 and Shannon index in the root surface of *S. superba* were higher than in the rhizosphere under all treatments (**Figures 6B**, **7B**).

Under N stress, the Chao1 index in the root surface and rhizosphere of *C. lanceolata* was higher than that of *S. superba* at the five N form ratios and the Shannon index in the root surface of *C. lanceolata* was higher than that of *S. superba* at the NH_4_^+^-N to NO_3_^−^-N ratio of 10:0 and 2:8 (**Figures 6C**, **7C**). Both the Chao1 and Shannon indices of the rhizosphere were higher in *C. lanceolata* than in *S. superba* (**Figures 6D**, **7D**).

### Structural composition of phylum levels in the root surface and rhizosphere bacterial communities of *C. lanceolata* and *S. superba* under N stress and N form ratios

**Figure 8:**
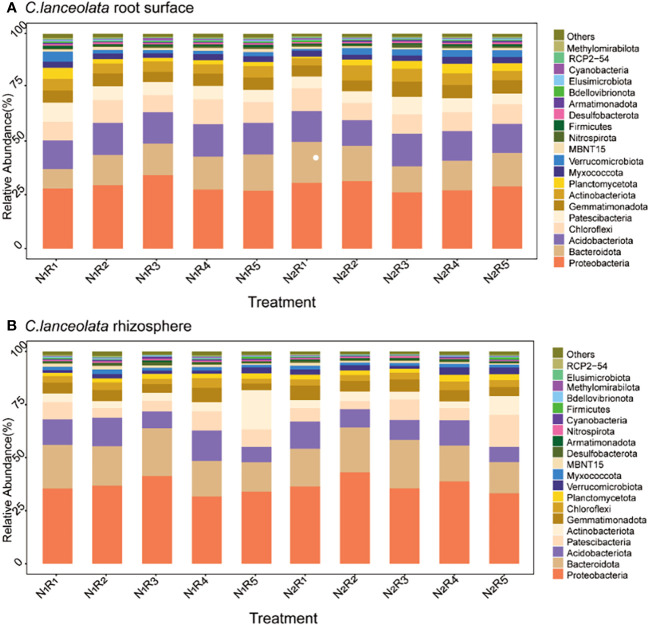
Relative abundance of the top 20 bacterial phyla in the root surface **(A)** and rhizosphere **(B)** of *C*. *lanceolata* at different N supply levels and N form ratio treatments.

At the phylum level, there were 20 most abundant bacteria in the root surface and rhizosphere of *C. lanceolata* (**Figures 8A, B**) with the same dominant phylum (>10%), Proteobacteria, Bacteroidota, and Acidobacteriota. Proteobacteria abundance in the root surface and rhizosphere of *C. lanceolata* was 23.12% higher than normal N supply at the NH_4_^+^-N to NO_3_^−^-N ratio of 8:2, and higher in the rhizosphere compared to in the root surface, while Acidobacteriota abundance was 9.94% higher than normal N supply at the NH_4_^+^-N to NO_3_^−^-N ratio of 2:8, and higher in the root surface compared to in the rhizosphere. Bacteroidota abundance in the root surface of *C. lanceolata* was 8.88% higher than the normal N supply at the NH_4_^+^-N to NO_3_^−^-N ratio of 5:5, while in the rhizosphere, it was 13.26% higher than the normal N supply at the NH_4_^+^-N to NO_3_^−^-N ratio of 10:0.

**Figure 9:**
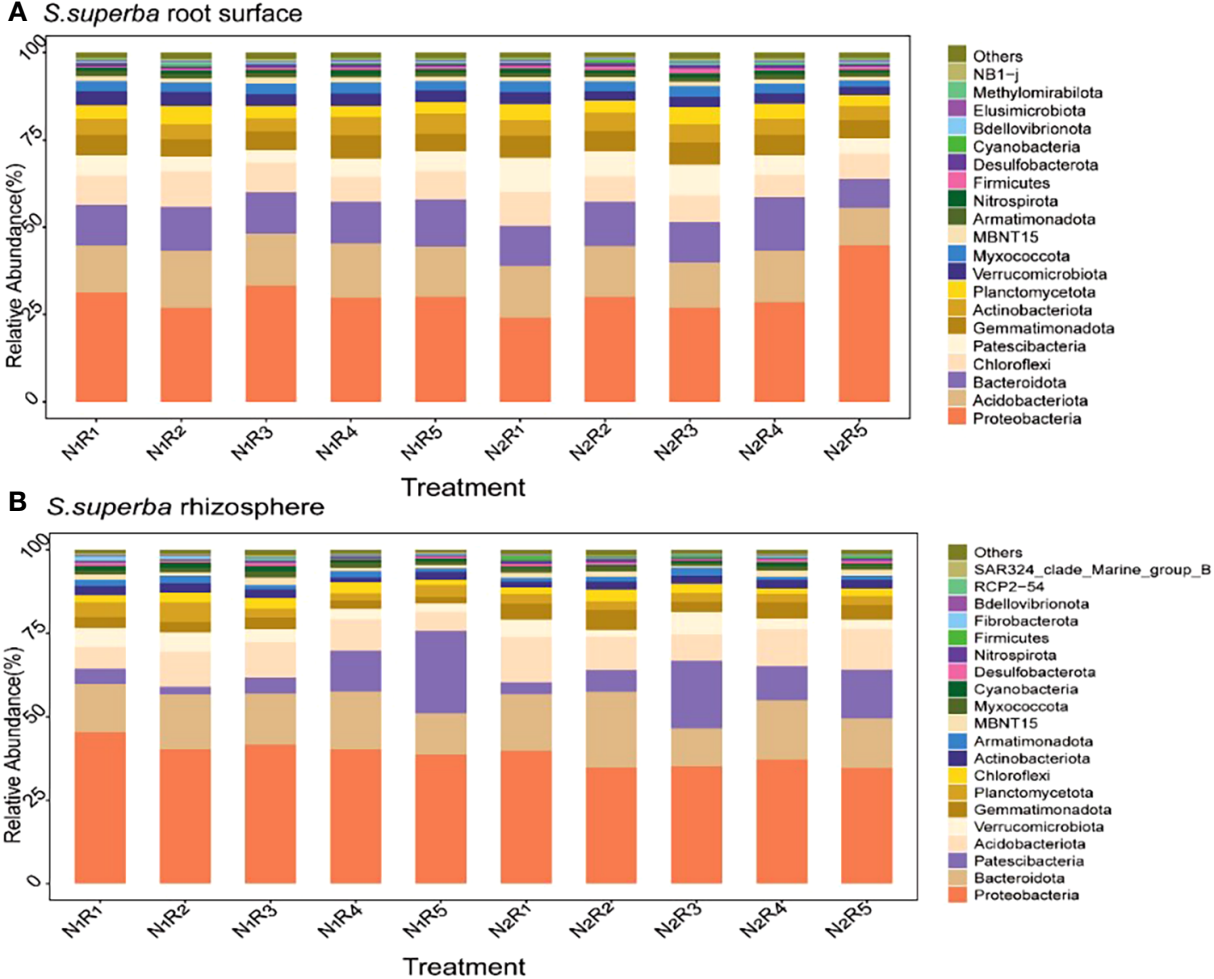
Relative abundance of the top 20 bacterial phyla in the root surface **(A)** and rhizosphere **(B)** of *S. superba* at different N supply levels and N form ratio treatments.

There were 20 most abundant bacteria in the root surface and rhizosphere of *S. superba* (**Figures 9A, B**). However, there were differences in dominant phyla between the root surface and rhizosphere, and the shared dominant phyla were Proteobacteria and Bacteroidota. The difference is that more Acidobacteriota accumulate in the root surface, while more Patescibacteria accumulate in the rhizosphere. The abundance of Acidobacteriota was 5.15%~25.10% higher than normal N supply at the other four N form ratios, except the NH_4_^+^-N to NO_3_^−^-N of 10:0 under N stress. The abundance of Patescibacteria was 66.59% higher than the normal N supply at the NH_4_^+^-N to NO_3_^−^-N ratio of 5:5. However, the abundance of Proteobacteria and Bacteroidota in *S. superba* showed different rules under different N supply levels and N form ratios. The relative abundance of Proteobacteria in the rhizosphere was higher than that in the root surface. Bacteroidota was higher in the rhizosphere than in the root surface, except with the NH_4_^+^-N to NO_3_^−^-N ratio of 5:5. The total abundance of the dominant phylum was higher in *C. lanceolata* than *in S. superba*. Different N levels and N form ratios had a greater effect on the bacterial abundance in the root surface and rhizosphere of the two tree species.

### Principal component analysis for bacterial communities in the root surface and rhizosphere of *C. lanceolata* and *S. superba* under N stress and N form ratios

**Figure 10:**
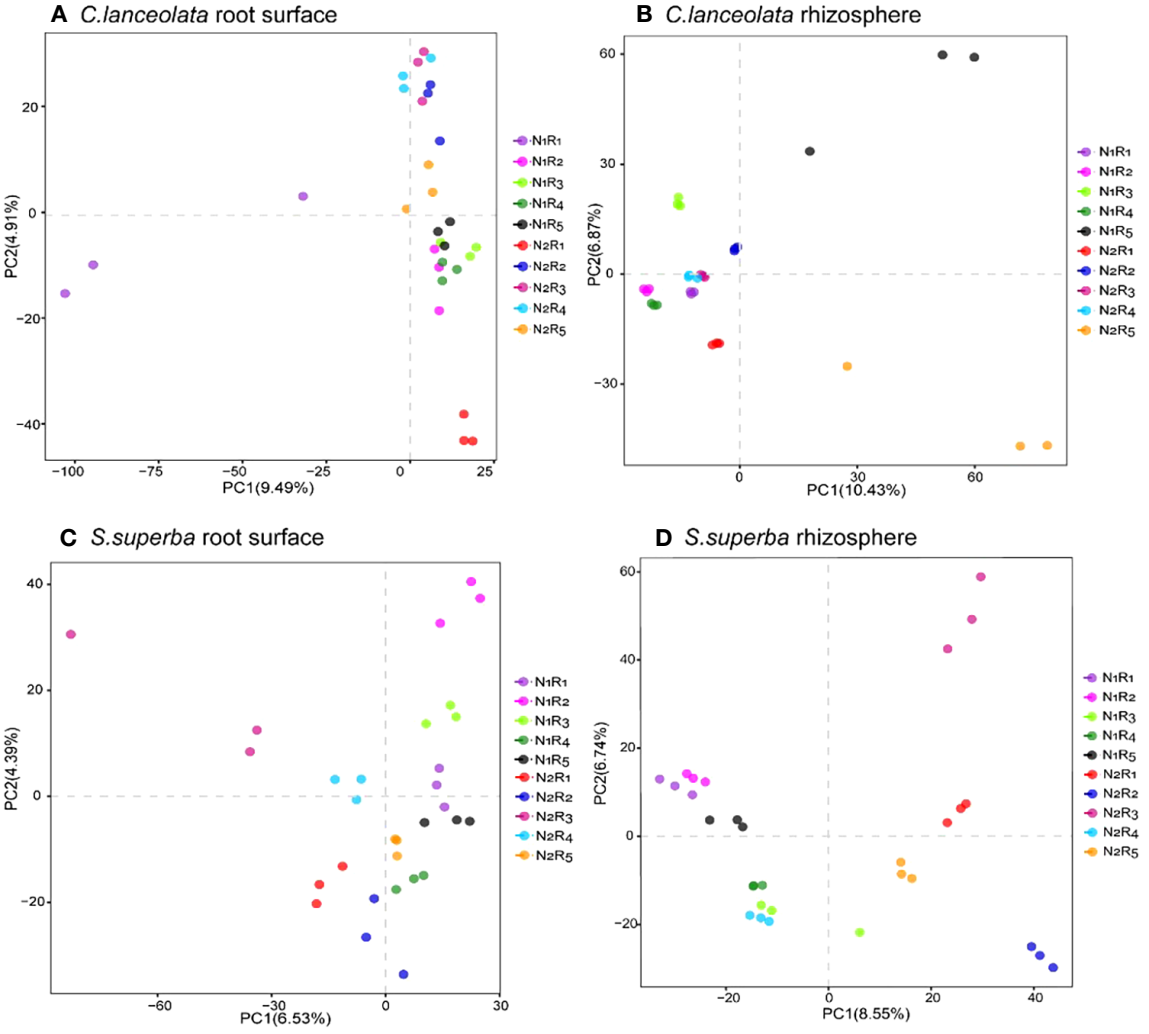
Partial least squares discriminant analysis grouped by the root surface of *C*. *lanceolata* **(A)**, by the rhizosphere of *C*. *lanceolata* **(B)**, by the root surface of *S. superba* **(C**), and by the rhizosphere of *S. superba* **(D)**.

The structural differences in bacterial communities between root surface and rhizosphere samples of *C. lanceolata* and *S. superba* were explored by downscaling (based on differences in OTUs). PC1 and PC2 explained 9.49% and 4.91% of the sample differences in the root surface of *C. lanceolata*, respectively (**Figure 10A**). The community structure of bacteria in the root surface showed a stronger segregation effect at the NH_4_^+^-N to NO_3_^−^-N ratio of 10:0 than the remaining four N form ratios, especially since the dispersion degree between repeats was larger under N stress. PC1 and PC2 explained 10.43% and 6.87% of the sample differences in the rhizosphere of *C. lanceolata*, respectively (**Figure 10B**). The NH_4_^+^-N to NO_3_^−^-N ratio of the 5:5 treatment had greater variation than the remaining four N form ratios at two N supply levels, which had greater dispersion than the remaining four treatments with the NH_4_^+^-N to NO_3_^−^-N ratio. PC1 and PC2 explained 6.53% and 4.39% of the sample differences in the root surface of *S. superba* (**Figure 10C**), while PC1 and PC2 explained 8.55% and 6.74% of the sample differences in the rhizosphere (**Figure 10D**), respectively. Under N stress, the proportions of five N form ratios in the root surface were evenly distributed on the same half-axis, which was just the opposite of that in the rhizosphere.

## Discussion

### Effects of N stress and N form ratios on the bacterial community composition in the root surface and rhizosphere of *C. lanceolata* and *S. superba*

Under nutrient stress, the interaction between plant and bacterial communities in the rhizosphere contributes to the efficiency of nutrient uptake by plants (Berendsen et al., 2012). Bacterial community and diversity are influenced by several factors, among which soil N supply level and N form ratio are considered to be important factors that directly or indirectly affect the soil bacterial community, especially bacteria with genes functional for N cycling (Szukics et al., 2009; Tang et al., 2016). In our study, the N supply level had a significant effect on the abundance but not on the diversity of bacteria in the root surface and rhizosphere of two tree species. Except for the interaction between the N supply level and tree species, the bacterial abundance and diversity were strongly influenced by the N form ratio, root compartment, tree species, and the interaction of the N supply level, N form ratio, root compartment, and tree species. Soil bacterial communities in *C. lanceolata* plantations varied with broadleaf mixed forest species and were influenced by the total N as well as ammonium N content of the soil (Liu et al., 2013). Each plant has a specific bacterial community. Studies on the model plant *Arabidopsis thaliana* showed that the root bacterial community consisted mainly of Proteobacteria, Actinobacteria, and Bacteroidota (Bulgarelli et al., 2012). In addition, the rhizosphere and endosphere typically contain Proteobacteria, Actinobacteria, and, to a lesser extent, Bacteroidota (Liu et al., 2017). In our study, the bacteria abundance higher than 1% in the root surface and rhizosphere of *C. lanceolata* and *S. superba* were completely consistent under the two N supply levels. Proteobacteria and Bacteroidota were common in the root surface and rhizosphere of two tree species, and their relative abundance was more than 10% in both cases, which indicates that the level of N supply did not change the bacterial composition. Proteobacteria and Bacteroidota are important components of the root surface and rhizosphere of two tree species.

Soil N is an extremely important factor, directly or indirectly, affecting the bacterial community. Numerous studies have shown that the addition of N results in significant changes in bacterial abundance, especially at the phylum level, and that bacterial abundance varies with the level of N supply (Beauregard et al, 2010; Wang et al, 2018). Proteobacteria is the most dominant bacterial phylum in soil (Lundberg et al., 2012), which belongs to the eutrophic bacterial community and is positively correlated with soil carbon availability. Most of the groups of Proteobacteria are N fixers and play an important role in the N cycle of the soil (Fierer et al, 2007; Lin et al., 2018). The relative abundance of co-nutrient taxa, mainly Proteobacteria and Bacteroidota, increased under high N treatment in a long-term N addition experiment (Fierer et al., 2012). Furthermore, the average relative abundance of Proteobacteria in the soil of young *C. lanceolata* forests increased after high N treatment (Hao et al., 2018; Guo P. P. et al., 2021). However, this is not the same as the present study, where there is some uncertainty about the effect of N addition on the soil bacterial community. This may be because high N addition can appropriately increase the relative abundance of the dominant bacterial phylum, as low N levels may not support the metabolic processes of bacterial growth (Liao et al., 2016). Proteobacteria abundance was not always higher under normal N supply than under N stress in this study. Although the abundance of Proteobacteria was highest in the root surface and rhizosphere communities of *C. lanceolata* and *S. superba*, it varied with N form ratios. The NH_4_^+^-N to NO_3_^−^-N ratio of 8:2 significantly increased the abundance of Proteobacteria in the root surface and rhizosphere of two tree species under N stress, and the abundance of Proteobacteria in both the root surface and rhizosphere of *C. lanceolata* was higher in this ratio than the other four N form ratios. It is also interesting to observe that the relative abundance of Proteobacteria was higher under N stress at all five N form ratios in the rhizosphere of *S. superba* than under normal N supply. This may be due to the greater ability of the soil bacterial community to utilize carbon sources under the low N treatment, and the high ammonium N supply enhances favorable survival conditions for the Proteobacteria.

The habitats of tree species had a significant effect on soil bacterial abundance. It was found that the composition of the bacterial community varied among the different species of deciduous trees (Wang et al., 2023). The relative abundance of Proteobacteria was lower in nutrient-poor *C. lanceolata* soils than in broadleaf forests (Yan et al., 2022). In our study, the abundance of Proteobacteria in *C. lanceolata* was lower than that in *S. superba*, which is consistent with the results of previous studies. It is hypothesized that broadleaf litter helps to increase soil organic matter content and provide energy for bacterial activity. In addition, Acidobacteriota played an equally important role in the bacterial communities in the root surface and rhizosphere of *C. lanceolata* and *S. superba*. The dominant phylum of bacteria in the rhizosphere of *S. superba*, which accumulated relatively more Acidobacteriota in the root surface and Patescibacteria in the rhizosphere, was more affected by N stress and N form ratio. The Acidobacteriota and Proteobacteria are the main phyla of *C. lanceolata* and *S. superba* plantations, with the Acidobacteria dominating (Cao et al., 2021; Wang et al., 2023). However, in our study, the abundance of Baobacteria in the surface and rhizosphere of two tree species was higher than that of Acidobacteriota. This may be because the soil where the study site is located is acidic, while the Acidobacteriota is acidophilic. Collectively, these results suggest that the relative abundance of the root surface and rhizosphere of *C. lanceolata* and *S. superba* were significantly affected by N supply level and nitrogen form ratio, especially the rhizosphere bacteria of *S. superba* were more sensitive to the changes.

### Effects of N stress and N form ratios on the abundance and diversity of bacteria in the root surface and rhizosphere of *C. lanceolata* and *S. superba*

Bacteria is an important component of the microbial community; its diversity reflects the overall dynamics of the community. Appropriate N addition provides a rich nutrient source for bacterial growth over a period of time, which contributes significantly to the abundance and diversity of bacterial communities, especially when mixed N addition significantly increases bacterial biomass (Xu et al., 2016; Song et al., 2022). Pau (1996) found that microbial abundance and biomass were higher under high N than in low N plots. In the present study, the bacterial abundance in the root surface and rhizosphere of *C. lanceolata* and *S. superba* was higher under normal N supply than under N stress in most treatments. Appropriate ratios of N forms could increase the abundance of bacterial communities and even significantly increase the abundance of bacterial communities in the root surface and rhizosphere under N stress, indicating that the N form ratio had a greater effect on soil bacterial abundance. It has been shown that the soil microbial communities have higher bacterial diversity under nitrogen deficiency stress. In a study on the effect of N stress on the structural characteristics of soil microbial communities in the rhizosphere of wheat, it was mentioned that N deficiency significantly increased the α-diversity of soil bacterial communities (Xiong et al., 2022). The Shannon diversity index combines the abundance and evenness of the community, with higher values indicating a greater diversity of the community. Yuan et al. (2012) found that high N inhibited the microbial biomass and abundance of *C. lanceolata*, and moderate low N treatment was beneficial to improve the microbiota Shannon diversity index and evenness index and promote microbial biomass. Kavamura et al. (2018) found that the Shannon index on the soil bacterial community of wheat was higher under low N conditions. This is consistent with the present study, where the bacterial diversity in both the root surface and rhizosphere of *C. lanceolata* and *S. superba* was higher under N stress than under normal N supply. The reason for this analysis may be due to the continuous accumulation of effective N in the soil, aggravating the acidification of the substrate and eventually leading to a decrease in the diversity of the bacterial community. However, this speculation needs further in-depth study and verification.

The diversity of microbiota showed a gradually decreasing trend from the rhizosphere to the inner boundary (Zgadzaj et al., 2016). In the present study, the abundance and diversity of bacteria in the root surface were higher than those in the rhizosphere of *C. lanceolata* and *S. superba* under N stress treatment, which was consistent with the results of previous studies and could be attributed to the secretions released from the root system. Root secretions are an important adaptive mechanism for plants to overcome N deficiency. For example, under N-limiting conditions, legumes release large amounts of flavonoids to attract N-fixing bacteria, thereby increasing soil N content. In some low-N soils, the relative contribution of root secretions to carbon input increases, providing essential nutrients for bacterial growth and metabolism and significantly increasing the biomass of soil microbia (Zhang and Mason, 2022). Furthermore, in this study, it was found that the bacterial diversity in the root surface of *C. lanceolata* was highest under single ammonium N treatment, while the bacteria in the root surface of *S. superba* was highest under single nitrate N treatment. This may be related to the different preferences of N uptake by different tree species in environments with heterogeneous N distribution. Previous studies suggested that *C. lanceolata* preferentially absorbed ammonium N and *S. superba* preferentially absorbed nitrate N (Zhang et al., 2013; Yan et al., 2019; Yan et al., 2020). It is worth noting that when N is taken up by plants in various N forms, it can cause changes in the pH of the rhizosphere soil. A study of bacterial communities in the rhizosphere showed that the soil pH had a high correlation with bacterial abundance and diversity. Closer to neutral soils, bacterial communities are more abundant, while the bacterial community abundance was lowest in acidic soils (Lauber et al., 2009). Therefore, the pH of the soil needs to be further studied. The enrichment effect of the rhizosphere on specific microorganisms was influenced by different tree species. Grayston and Prescott (2005) found that the relative abundance of soil microorganisms was high when the leaves were high in calcium for four Canadian tree species. Different tree species respond differently to soil nutrients, which is an important factor influencing soil bacterial communities. Zhang Zehao found that short-term N addition helped increase the diversity of bacterial communities in salt-tolerant plants (Zhang et al., 2023). The bacterial communities and diversity of different tree species were positively correlated with total soil nitrogen in the South Asian tropics. However, differences in bacterial diversity among tree species were not apparent (Qin et al., 2021). In the present study, the relative abundance of bacteria in the root surface and rhizosphere of *C. lanceolata* was significantly higher than that of *S. superba*, presumably by altering the quality of plant-derived carbon, which may influence the microbial community. In conclusion, the diversity of bacteria was significantly increased by an appropriate N form ratio under N stress. Moreover, the important role of root surface bacteria in influencing plant nitrogen cannot be ignored.

## Conclusion

In the present study, we explored the bacterial community composition and diversity on the root surface and rhizosphere of *C. lanceolata* and *S. superba* under different N supply levels and N form ratios. N stress and N form ratios had significant effects on the bacterial community diversity on the root surface and rhizosphere of *C. lanceolata* and *S. superba*, and altered the abundance of the dominant phylum, but did significantly change the composition of the bacterial community in the two tree species. Under N stress, the bacterial community diversity in the root surface of two tree species was higher than that in the rhizosphere. The bacterial diversity in the root surface of *C. lanceolata* was highest under the complete supply of ammonium N, and the bacterial diversity in the root surface of *S. superba* was highest under the complete supply of nitrate N, and a homogeneous supply of ammonium and nitrate N can significantly reduce the bacteria diversity in the rhizosphere of two tree species. The bacterial community in different tree species and different root compartments responded differently to the N supply level. A reasonable ammonium-nitrate N form ratio plays a key role in the root surface and rhizosphere under the N-limited soil.

## Data availability statement

The datasets presented in this study can be found in online repositories. The names of the repository/repositories and accession number(s) can be found below: https://www.ncbi.nlm.nih.gov/, accession number PRJNA986907.

## Author contributions

Conceptualization, YW and XY. Methodology and formal analysis, YW, XL, and XQ. Investigation, YW, XL, and XQ. Data curation, YW, XL, and XQ. Writing original draft preparation, YW and XY. Writing review and editing, YW and XY. Supervision, XY. Funding acquisition, XY. All authors contributed to the article and approved the submitted version.

## References

[B5] Bai B. Liu W. D. Qiu X. Y. Zhang J. Zhang J. Y. Bai Y. (2022). The root microbiome: Community assembly and its contributions to plant fitness. J. Integr. Plant Biol. 64, 230–243. doi: 10.1111/jipb.13226 35029016

[B1] Beauregard M. S. Hamel C. Atul-Nayyar St-Arnaud M. (2010). Long term phosphorus fertilization impacts soil fungal and bacterial diversity but not AM fungal community in Alfalfa. Microb. Ecol. 59, 379–389. doi: 10.1007/s00248-009-9583-z 19756847

[B2] Berendsen R. L. Pieterse C. M. J. Bakker P. A. H. M. (2012). The rhizosphere microbiome and plant health. Trends Plant Sci. 17, 478–486. doi: 10.1016/j.tplants.2012.04.001 22564542

[B3] Berthrong S. T. Yeager C. Gallegos-Graves L. Steven B. Eichorst S. A. Jackson R. B.. (2014). Nitrogen fertilization has a stronger effect on soil nitrogen-fixing bacterial communities than elevated atmospheric CO_2_ . Appl. Environ. Microbiol. 80, 3103–3112. doi: 10.1128/AEM.04034-13 24610855 PMC4018900

[B4] Bulgarelli D. Rott M. Schlaeppi K. Themaat E. V.L. Ahmadinejad N. Assenza F.. (2012). Revealing structure and assembly cues for *Arabidopsis* root-inhabiting bacterial microbiota. Nature. 488, 91–95. doi: 10.1038/nature11336 22859207

[B7] Cao S. Pan F. Lin G. G. Zhang Y. L. Zhou C. D. Liu B.. (2021). Changes of soil bacterial structure and soil enzyme activity in Chinese fir forest of different ages. Acta Ecol. Sin. 41, 1846–1856. doi: 10.5846/stxb202004010772

[B6] Carvalho P. A. Oliveira L. E. Sodek L. Carvalho J. N. D. (2015). Nitrogen metabolism in the roots of rubber tree (‘*Hevea brasiliensis*’) plants supplied with nitrate or ammonium as nitrogen source during hypoxia. Aust. J. Crop Sci. 9, 1278–1285.

[B8] Chen S. M. Waghmode T. R. Sun R. B. Kuramae E. Hu C. S. Liu B. B. (2019). Root-associated microbiomes of wheat under the combined effect of plant development and nitrogen fertilization. Microbiome. 7, 136. doi: 10.1186/s40168-019-0750-2 31640813 PMC6806522

[B9] Choudhary D. K. Kasotia A. Jain S. Vaishnav A. Kumari S. Sharma K. P.. (2016). Bacterial mediated tolerance and resistance to plants under abiotic and biotic stresses. J. Plant Growth Regul. 35, 276–300. doi: 10.1007/s00344-015-9521-x

[B10] Dini-Andreote F. Pylro V. Baldrian P. van Elsas J. D. Salles J. F. (2016). Ecological succession reveals potential signatures of marine-terrestrial transition in salt marsh fungal communities. ISME J. 10, 1984–1997. doi: 10.1038/ismej.2015.254 26824176 PMC5029165

[B11] Edgar R. C. (2013). UPARSE: highly accurate OTU sequences from microbial amplicon reads. Nat. Methods 10, 996–998. doi: 10.1038/nmeth.2604 23955772

[B14] Fei Y. C. Wu Q. Z. Lu J. Ji C. S. Zheng H. Cao S. J.. (2020). Effects of undergrowth vegetation management measures on the soil bacterial community structure of large diameter timber plantation of *Cunninghamia lanceolata* . Chin. J. Appl. Ecol. 31, 407–416. doi: 10.13287/j.1001-9332.202002.035 32476332

[B13] Fierer N. Bradford M. A. Jackson R. B. (2007). Toward an ecological classification of soil bacteria. Ecology. 88, 1354–1364. doi: 10.1890/05-1839 17601128

[B12] Fierer N. Lauber C. L. Ramirez K. S. Zaneveld J. Bradford M. A. Knight B. (2012). Comparative metagenomic, phylogenetic and physiological analyses of soil microbial communities across nitrogen gradients. ISME J. 6, 1007–1017. doi: 10.1038/ismej.2011.159 22134642 PMC3329107

[B15] Grayston S. J. Prescott C. E. (2005). Microbial communities in forest floors under four tree species in coastal British Columbia. Soil Biol. Biochem. 37, 1157–1167. doi: 10.1016/j.soilbio.2004.11.014

[B16] Guo J. Wu Y. Q. Wu X. H. Ren Z. Wang G. B. (2021). Soil bacterial community composition and diversity response to land conversion is depth-dependent. Glob Ecol. Conserv. 32, e01923. doi: 10.1016/j.gecco.2021.e01923

[B17] Guo P. P. Huang X. R. Wu W. W. Zheng L. L. Fang X. Yi Z. G. (2021). Effects of different nitrogen application methods and levels on soil bacterial communities of *Pinus massoniana* and *Schima superba* seedling roots. Acta Ecol. Sin. 41, 149–161. doi: 10.5846/stxb201907071429

[B18] Hao Y. Q. Xie L. Chen Y. M. Tang C. D. Liu X. F. Lin W. S.. (2018). Effects of nitrogen deposition on diversity and composition of soil bacterial community in a subtropical *Cunninghamia lanceolata* plantation. Chin. J. Appl. Ecol. 29, 53–58. doi: 10.13287/j.1001-9332.201801.034 29692012

[B19] Jing X. Sanders N. Shi Y. Chu H. Y. Classen A. Zhao K.. (2015). The links between ecosystem multifunctionality and above- and belowground biodiversity are mediated by climate. Nat. Commun. 6, 8159. doi: 10.1038/ncomms9159 26328906 PMC4569729

[B20] Kavamura V. N. Hayat R. Clark I. M. Rossmann M. Mendes R. Hirsch P. R.. (2018). Inorganic nitrogen application affects both taxonomical and predicted functional structure of wheat rhizosphere bacterial communities. Front. Microbiol. 9, 1074. doi: 10.3389/fmicb.2018.01074 29896167 PMC5986887

[B21] Kiba T. Krapp A. (2016). Plant nitrogen acquisition under low availability: regulation of uptake and root architecture. Plant Cell Physiol. 57, 707–714. doi: 10.1093/pcp/pcw052 27025887 PMC4836452

[B22] Lauber C. L. Hamady M. Knight R. Fierer N. (2009). Pyrosequencing-based assessment of soil pH as a predictor of soil bacterial community structure at the continental scale. Appl. Environ. Microbiol. 75, 5111–5120. doi: 10.1128/AEM.00335-09 19502440 PMC2725504

[B24] Li Y. L. Tremblay J. Bainard L. D. Cade-Menun B. Hamel C. (2020). Long-term effects of nitrogen and phosphorus fertilization on soil microbial community structure and function under continuous wheat production. Environ. Microbiol. 22, 1066–1088. doi: 10.1111/1462-2920.14824 31600863

[B25] Liang H. Y. Wang L. D. Wang Y. R. Quan X. Q. Li X. Y. Xiao Y. N.. (2022). Root development in *Cunninghamia lanceolata* and *Schima superba* seedlings expresses contrasting preferences to nitrogen forms. Forests. 13, 2085. doi: 10.3390/f13122085

[B26] Liao N. Li Q. Zhang W. Zhou G. W. Ma L. J. Min W.. (2016). Effects of biochar on soil microbial community composition and activity in drip-irrigated desert soil. Eur. J. Soil Biol. 72, 27–34. doi: 10.1016/j.ejsobi.2015.12.008

[B27] Lin B. S. Fan J. L. Song Z. Z. Zhang L. L. Zhang Y. L. Lin Z. X. (2018). Endophytic diazotrophs composition of Pennisetum sp. at different growth stages. Microbiol. China. 45, 1479–1490. doi: 10.13344/j.microbiol.china.170730

[B29] Liu H. W. Carvalhais L. C. Crawford M. H. Singh E. Dennis P. J. Pieterse C. M. J.. (2017). Inner plant values: diversity, colonization and benefits from endophytic bacteria. Front. Microbiol. 8, 1–17. doi: 10.3389/fmicb.2017.02552 29312235 PMC5742157

[B23] Liu L. Xu M. K. Wang S. L. Zhang Q. R. Wang N. Pan H. Q.. (2013). Effect of different *Cunninghamia lanceolata* plantation soil qualities on soil microbial community structure. Acta Ecologica Sinica. 33, 4692–4706. doi: 10.5846/stxb201205020628

[B28] Liu S. R. Yang Y. J. Wang H. (2018). Development strategy and management countermeasures of planted forests in China: transforming from timber-centered single objective management towards multi-purpose management for enhancing quality and benefits of ecosystem services. Acta Ecol. Sin. 38, 1–10. doi: 10.5846/stxb201712072201

[B30] Lundberg D. S. Lebeis S. L. Paredes S. H. Yourstone S. Gehring J. Malfatti S.. (2012). Defining the core *Arabidopsis thaliana* root microbiome. Nature. 488, 86–90. doi: 10.1038/nature11237 22859206 PMC4074413

[B31] Miller G. E. Engen P. A. Gillevet P. M. Shaikh M. Sikaroodi M. Forsyth C. B.. (2016). Lower neighborhood socioeconomic status associated with reduced diversity of the colonic microbiota in healthy adults. PloS One 11, e0148952. doi: 10.1371/journal.pone.0148952 26859894 PMC4747579

[B32] Nacry P. Bouguyon E. Gojon A. (2013). Nitrogen acquisition by roots: physiological and developmental mechanisms ensuring plant adaptation to a fluctuating resource. Plant Soil. 370, 1–29. doi: 10.1007/s11104-013-1645-9

[B33] Pau J. W. (1996). Soil microbial biomass Co N mineralization, and N uptake by corn in dairy cattle slurry- and urea-amended soils. Can. J. Soil Sci. 76, 469–472. doi: 10.4141/cjss96-058

[B34] Qin X. H. Liang Y. Chen C. F. Qin L. (2021). Effects of different tree species plantations on soil bacterial community diversity in south subtropical China. For. Res. 34, 120–127. doi: 10.13275/j.cnki.lykxyj.2021.04.014

[B35] Rognes T. Flouri T. Nichols B. Quince C. Mahé F. (2016). VSEARCH: a versatile open source tool for metagenomics. PeerJ. 4, e2584. doi: 10.7717/peerj.2584 27781170 PMC5075697

[B1000] Song G. Li X. J. Wang Q. C. Lyu M. K. Xie J. S. He J. Z.. (2022). Responses of soil microbial biomass and carbon source utilization to simulated nitrogen deposition and drought in a Cunninghamia lanceolata plantation. Chinese J. Appl. Ecol. 33, 2388–2396. doi: 10.13287/j.1001-9332.202209.016 36131654

[B36] Sun M. H. Lu X. P. Cao X. J. Li J. Xiong J. Xie S. X. (2015). Effect of different nitrogen forms on root growth and dynamic kinetics characteristics for. Citrus sinensis × Poncirus trifoliata. Scientia Silvae Sinicae. 51, 113–120. doi: 10.11707/j.1001-7488.20151214

[B37] Suo P. H. Du D. J. Wang Y. Z. Hu Y. L. Liu X (2019). Effects of successive rotation Chinese fir plantations on soil nitrogen content and soil enzyme activities related to nitrogen transformation. J. For Environ. Sci. 39, 113–119. doi: 10.13324/j.cnki.jfcf.2019.02.001

[B38] Szukics U. Hackl E. Zechmeister-Boltenstern S. Sessitsch A. (2009). Contrasting response of two forest soils to nitrogen input: rapidly altered NO and N_2_O emissions and nirK abundance. Biol. Fertil Soils. 45, 855–863. doi: 10.1007/s00374-009-0396-5

[B39] Tang Y. Q. Zhang X. Y. Li D. D. Wang H. M. Chen F. S. Fu X. L.. (2016). Impacts of nitrogen and phosphorus additions on the abundance and community structure of ammonia oxidizers and denitrifying bacteria in Chinese fir plantations. Soil Biol. Biochem. 103, 284–293. doi: 10.1016/j.soilbio.2016.09.001

[B40] Tian D. L. Shen Y. Kang W. X. Xiang W. H. Yan W. D. Deng X. W. (2011). Characteristics of nutrient cycling in first and second rotations of Chinese fir plantations. Acta Ecol. Sin. 31, 5025–5032.

[B41] Veresoglou S. D. Chen B. Rillig M. C. (2012). Arbuscular mycorrhiza and soil nitrogen cycling. Soil Biol. Biochem. 46, 53–62. doi: 10.1016/j.soilbio.2011.11.018

[B42] Wang L. Y. Sun Y. Y. Li J. Tigabu M. Xu Q. L. Ma X. Q.. (2023). Rhizosphere soil nutrients and bacterial community diversity of four broad-leaved trees planted under Chinese fir stands with different stocking density levels. Front. For. Glob. Change 6, 1135692. doi: 10.3389/ffgc.2023.1135692

[B43] Wang Q. Wang C. Yu W. W. Turak A. Chen D. W. Huang Y.. (2018). Effects of nitrogen and phosphorus inputs on soil bacterial abundance, diversity, and community composition in Chinese fir plantation. Front. Microbiol. 9, 1453. doi: 10.3389/fmicb.2018.01543 30072961 PMC6060263

[B44] Wu P. F. Ma X. Q. Tigabu M. Wang C. Liu A. Q. Odén P. C.. (2011). Root morphological plasticity and biomass production of two Chinese fir clones with high phosphorus efficiency under low phosphorus stress. Can. J. For Res. 41, 228–234. doi: 10.1139/X10-198

[B45] Xiao H. Y. Liu B. Yu Z. P. Wan X. H. Sang C. P. Zhou F. W.. (2017). Seasonal dynamics of soil mineral nitrogen pools and nitrogen mineralization rate in different forests in subtropical China. Chin. J. Appl. Ecol. 28, 730–738. doi: 10.13287/j.1001-9332.201703.036 29740997

[B46] Xiong Y. Zheng L. Shen R. F. Lan P. (2022). Effects of nitrogen deficiency on microbial community structure in rhizosphere soil of wheat. Acta Petrol Sin. 59, 218–230. doi: 10.11766/trxb202005080225

[B47] Xu K. Wang C. M. Zhang Y. Yang X. T. Liu W. M. (2016). Effect of simulated atmospheric nitrogen deposition on soil microbial community structure in a temperate forest. Chin. J. Ecol. 35, 2676–2683. doi: 10.13292/j.1000-4890.201610.012

[B50] Yan X. L. Hu W. J. Ma Y. F. Huo Y. F. Wang T. Ma X. Q. (2020). Nitrogen uptake preference of *Cunninghamia lanceolata*, *Pinus massoniana*, and *Schima superba* under heterogeneous nitrogen supply environment and their root foraging strategies. Scientia Silvae Sinicae. 56, 1–11. doi: 10.11707/j.1001-7488.20200201

[B51] Yan S. X. Liu M. Liu C. X. Zhao M. L. Qiu W. Gu J. Y.. (2022). Soil microbial diversity is higher in pure stands of moso bamboo than in pure stands of Chinese fir. Acta Petrol Sin. 59, 1704–1717. doi: 10.11766/trxb202107190169

[B49] Yan X. L. Ma X. Q. (2021). Responses of root morphology and seedling growth in three tree species to heterogeneous supplies of ammonium and nitrate. For Ecol. Manage. 479, 118538. doi: 10.1016/j.foreco.2020.118538

[B48] Yan X. L. Wang C. Ma X. Q. Wu P. (2019). Root morphology and seedling growth of three tree species in southern China in response to homogeneous and heterogeneous phosphorus supplies. Trees. 33, 1283–1297. doi: 10.1007/s00468-019-01858-x

[B52] Yuan Y. H. Fan H. B. Li H. X. Liu W. F. Shen F. F. Guo H. B. (2012). Effects of simulated nitrogen deposition on soil microorganism in a Chinese fir plantation. Scientia Silvae Sinicae. 48, 8–14. doi: 10.11707/j.1001-7488.20120902

[B53] Zeng J. Liu X. J. Song L. Lin X. G. Zhang H. Y. Shen C. C.. (2016). Nitrogen fertilization directly affects soil bacterial diversity and indirectly affects bacterial community composition. Soil Biol. Biochem. 92, 41–49. doi: 10.1016/j.soilbio.2015.09.018

[B55] Zgadzaj R. Garrido-Oter R. Jensen. D. B. Koprivova A. Schulze-Lefert P. Radutoiu S. (2016). Root nodule symbiosis in *Lotus japonicus* drives the establishment of distinctive rhizosphere, root, and nodule bacterial communities. Proc. Natl. Acad. Sci. U.S.A. 113, 7996–8005. doi: 10.1073/pnas.1616564113 PMC5150415 27864511

[B59] Zhang Y. Bai S. B. (2003). Effects of nitrogen forms on nutrient uptake and growth of trees. Chin. J. Appl. Ecol. 14, 2044–2048. 14997674

[B56] Zhang N. Y. Guo R. Song P. Guo J. X. Gao Y. Z. (2013). Effects of warming and nitrogen deposition on the coupling mechanism between soil nitrogen and phosphorus in Songnen Meadow Steppe, northeastern China. Soil Biol. Biochem. 65, 96–104. doi: 10.1016/j.soilbio.2013.05.015

[B57] Zhang W. Mason G. A. (2022). Modulating the rhizosphere microbiome by altering the cocktail of root secretions. Plant Physiol. 188, 12–13. doi: 10.1093/plphys/kiab480 35051291 PMC8774711

[B54] Zhang J. G. Sheng W. T. Xiong Y. Q. Wan X. R. (2006). Effects of fertilization on soil nutrient content of potted Chinese fir seedling. Scientia Silvae Sinicae. 42, 44–50. doi: 10.11707/j.1001-7488.20060408

[B1001] Zhang Z. H. Li T. Shao P. S. Sun J. K. Xu W. J. Zhao Y. H. (2023). Effects of short-term nitrogen addition on rhizosphere and bulk soil bacterial community structure of three halophytes in the Yellow River Delta. Land Degrad. Dev. 34, 3281–3294. doi: 10.1002/ldr.4683

[B60] Zhao C. Peng S. Ruan H. H. Zhang Y. K. (2015). Effects of nitrogen deposition on soil microbes. J. Nanjing Forestry Univ. (Natural Sci. Edition). 58, 149–155. doi: 10.3969/j.issn.1000-2006.2015.03.027

[B58] Zhou X. Fu Y. L Zhou L. Y. Li B. Luo Y. Q. (2013). An imperative need for global change research in tropical forests. Tree Physiol. 33, 903–912. doi: 10.1093/treephys/tpt064 24128847

